# Gender disparities among Brazil's top 2% of influential scientists for career-long impact

**DOI:** 10.1590/1516-3180.2025.3131.03022026

**Published:** 2026-09-21

**Authors:** Jullyana de Souza Siqueira Quintans, Adriana Gibara Guimarães, Lucindo Quintans-Júnior, Adriano Antunes de Souza Araújo, Victor Santana Santos, Paulo Ricardo Martins-Filho

**Affiliations:** I Professor, Laboratório de Neurociências e Ensaios Farmacológicos (Lanef), Departamento de Ciências Fisiológicas, Universidade Federal de Sergipe, São Cristóvão (SE), Brazil.; II Professor, Programa de Pós-Graduação em Farmácia, Departamento de Farmácia, Universidade Federal de Sergipe, São Cristóvão (SE), Brazil.; III Professor, Laboratório de Neurociências e Ensaios Farmacológicos (Lanef), Departamento de Ciências Fisiológicas, Universidade Federal de Sergipe, São Cristóvão (SE), Brazil.; IV Professor, Laboratório de Ensaios Farmacêuticos e Toxicidade (LeFT), Departamento de Farmácia, Universidade Federal de Sergipe, São Cristóvão (SE), Brazil.; V Professor, Núcleo de Epidemiologia e Saúde Pública (Nesp), Departamento de Medicina, Universidade Federal de Sergipe, Lagarto (SE), Brazil.; VI Professor, Laboratório de Patologia Investigativa, Departamento de Educação em Saúde, Universidade Federal de Sergipe, Lagarto (SE), Brazil.

Dear Editor,

The ranking of the world's top 2% most influential scientists, published by Stanford University, has gained recognition for its comprehensive methodology in evaluating scientific impact.^1^ Developed by Professor John P.A. Ioannidis and his team, this ranking employs a composite metric based on data from the Scopus database to analyze scientists’ contributions within their respective fields.

Researchers are classified both for their career-long impact and recent performance, which are determined by a score composed of six citation indicators. These include total impact (number of citations, Hirsch index), coauthorship adjustment (Schreiber's Hm index), and authorship order metrics (total citations in papers as single author; single or first author; single, first, or last author).^2^ Although these metrics have limitations, Stanford University's ranking serves as a reference for assessing scientific output and impact, and identifying disparities that can guide policies that promote equity and scientific advancement.

In recent decades, Brazil has emerged as a significant contributor to global scientific production, despite historical challenges in science funding and regional inequalities. Female Brazilian researchers face barriers in opportunities, recognition, and career advancement, leading to their underrepresentation in prominent positions and in scientific rankings such as that of Stanford. This bias undermines gender equity and may limit innovation and diversity in Brazilian research.

Based on the ranking published on September 16, 2024, we extracted career-long data for researchers affiliated with Brazilian institutions, categorizing them by gender and geographic region (Central-West, Northeast, North, Southeast, and South).^1^ Using descriptive analysis, we calculated the frequency of male and female scientists per region and displayed this distribution on a map.

Out of 217,093 scientists listed, 1,077 were affiliated with Brazilian institutions. Four entries were excluded due to duplication or inconsistencies, resulting in 1,073 (0.5%) scientists classified as the most influential for career-long impact. Among these, 892 (83.1%) were male and 181 (16.9%) were female scientists. This discrepancy was observed across all regions, with the proportion of female scientists ranging from 18.3% in the South to 10% in the North (Figure 1).

**Figure 1:**
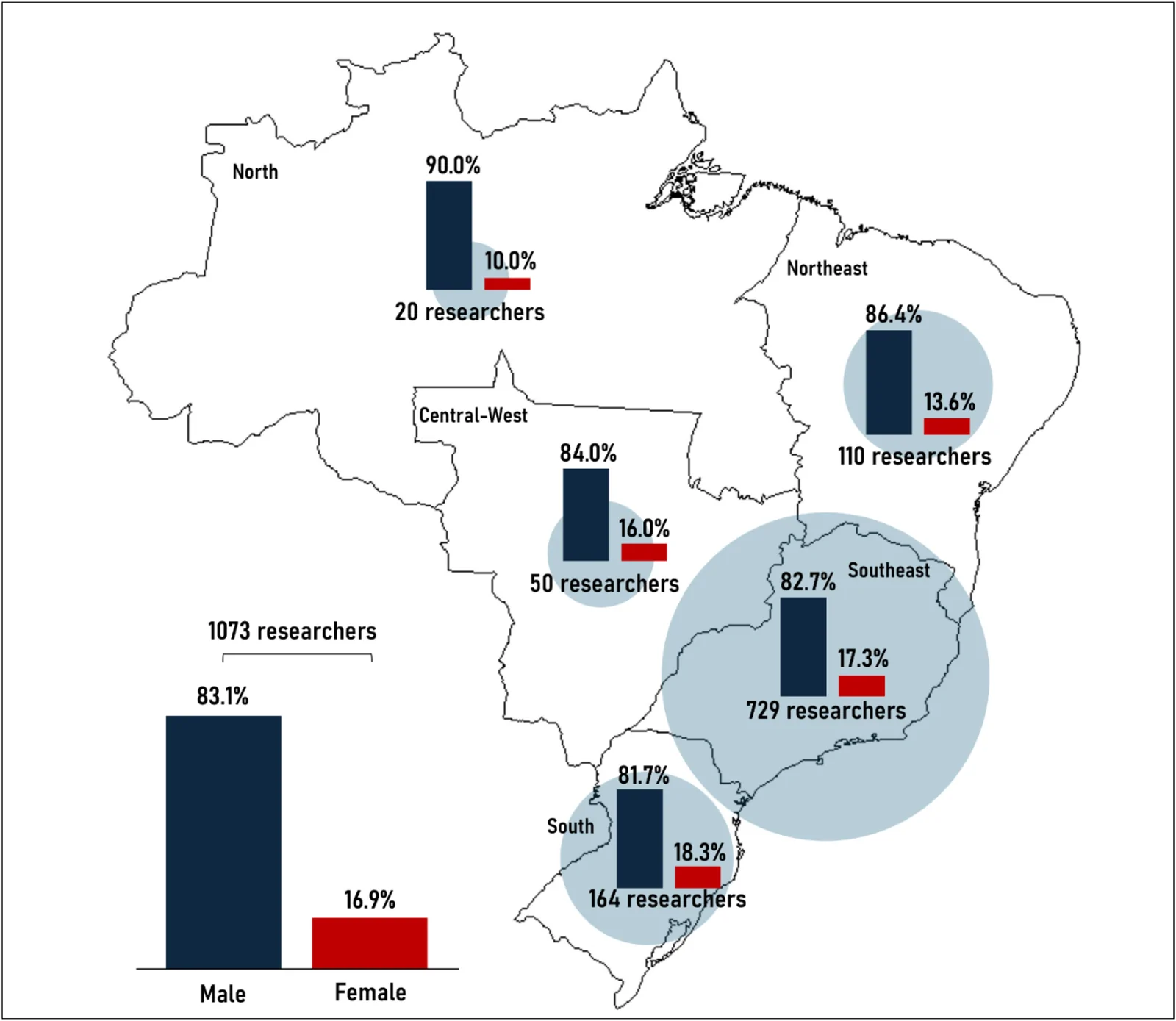
Distribution of male and female scientists in Brazil's top 2% of the most influential researchers by region.

Gender disparity in Brazil stems from historical, cultural, social, and institutional factors that collectively create barriers to equity in science. Women face challenges regarding funding, collaboration networks, and access to leadership positions.^3^ The predominance of men in decision-making roles at universities and funding agencies limits opportunities for women's advancement. Furthermore, segregation in certain fields, inequalities in authorship roles, and reduced recognition in peer review may affect knowledge production, acknowledgment of women's contributions, and visibility in citation-based rankings.^4^

Although the proportion of female scientists in prominent positions is low nationwide, discrepancies are greater in less affluent regions such as the North and Northeast, where funding disparities exacerbate regional inequalities. In 2023, of the R$ 5 billion (US$ 0.9 billion) allocated by the National Fund for Scientific and Technological Development (FNDCT), the main science funding body in Brazil, only 4% was directed to institutions in the North and Northeast, whereas 93% was concentrated in the South and Southeast.^5^ This imbalance makes it more difficult for women in underfunded regions to advance. Female researchers in Brazil have fewer research grants, published articles, and international collaborations,^6^ especially in resource-scarce regions. The coronavirus disease 2019 pandemic further accentuated these disparities, with countries such as Brazil showing a larger decline in women's publication rates.^7^

Despite advances in recent decades, gender inequality in academia remains a problem that requires effective policies and practices. Differences in career progression are impacted by factors such as maternity interruptions, discrimination, and family responsibilities. These factors create unfair competition between genders, and cultural change can take generations to materialize, leading to cumulative effects and losses in scientific development. To address this disparity, it is essential for universities, research institutes, and funding agencies to implement gender equity policies, with clear goals to increase female participation at all academic levels.

Educational programs that inspire young girls to pursue science careers, challenging gender stereotypes, are crucial. Funding initiatives for women-led projects, especially in less favored regions, can help mitigate disparities. Ensuring transparency and recognition of contributions in publications can reduce biases in authorship attribution. Raising awareness about unconscious biases and stereotypes in academia, providing parental leave, offering flexible working hours, and making childcare services available in universities and research institutes are also essential. Regular disclosure of gender-disaggregated data on funding, publications, promotions, and other metrics can support monitoring and accountability efforts.

Addressing the identified gender disparities requires that evidence-based policies and institutional reforms be implemented to promote gender equity at all academic levels. Such measures are essential not only for fairness but also to enhance the quality, innovation, and global competitiveness of scientific research.

## Data Availability

Data supporting the findings of this study are available upon request from the corresponding author, Paulo Ricardo Martins-Filho.
